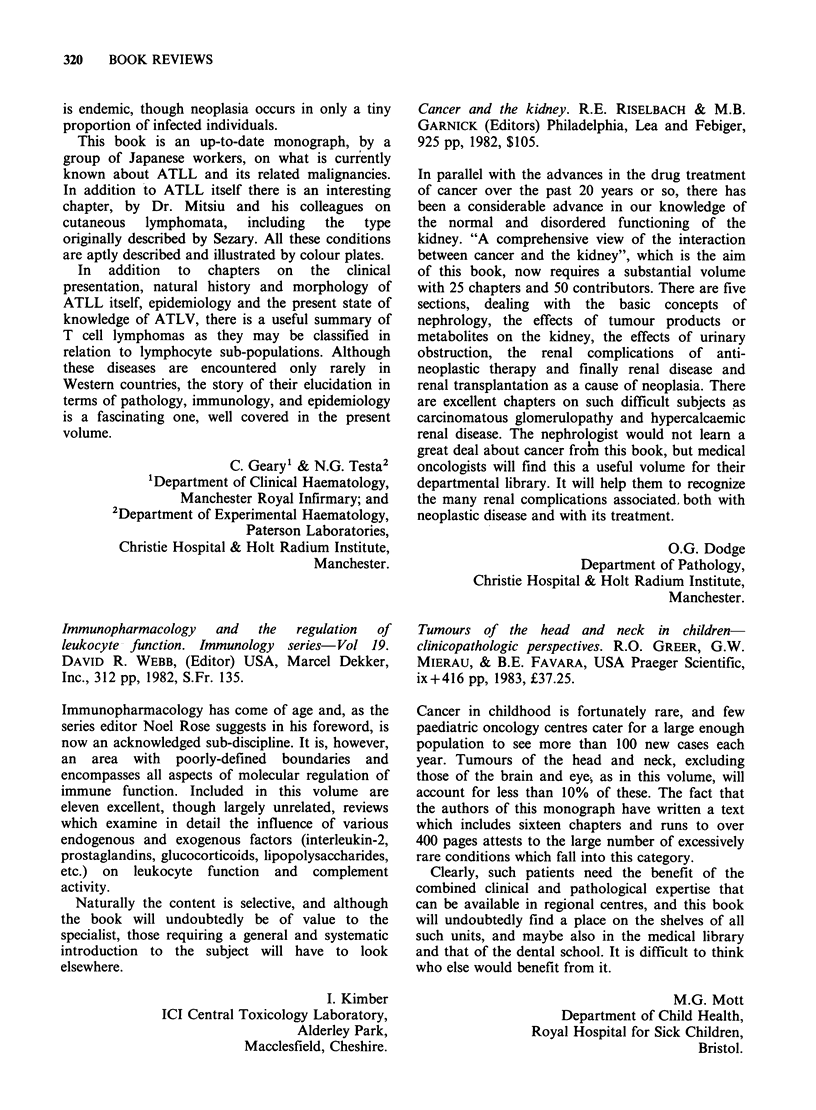# Immunopharmacology and the regulation of leukocyte function. Immunology series—Vol 19

**Published:** 1983-08

**Authors:** I. Kimber


*Immunopharmacology and the regulation of leukocyte function. Immunology series—Vol 19.* DAVID R. WEBB, (Editor) USA, Marcel Dekker, Inc., 312 pp, 1982, S.Fr. 135.

Immunopharmacology has come of age and, as the series editor Noel Rose suggests in his foreword, is now an acknowledged sub-discipline. It is, however, an area with poorly-defined boundaries and encompasses all aspects of molecular regulation of immune function. Included in this volume are eleven excellent, though largely unrelated, reviews which examine in detail the influence of various endogenous and exogenous factors (interleukin-2, prostaglandins, glucocorticoids, lipopolysaccharides, etc.) on leukocyte function and complement activity.

Naturally the content is selective, and although the book will undoubtedly be of value to the specialist, those requiring a general and systematic introduction to the subject will have to look elsewhere.

I. Kimber
ICI Central Toxicology Laboratory,
Alderley Park,
Macclesfield, Cheshire.